# Supplementary material for: The evaluating prescription opioid changes in veterans (EPOCH) study: Design, survey response, and baseline characteristics
Source: PLoS One. 2020 Apr 22;15(4):e0230751. doi: 10.1371/journal.pone.0230751 (PMC7176145; doi:10.1371/journal.pone.0230751)
Supplement: S1 Table — (DOCX) [file pone.0230751.s001.docx]

**S1 Table. Opioid formulations and dosage conversion factors**

| **Qualifying opioid analgesics indicated for chronic pain treatment** | | |
| --- | --- | --- |
| **Opioid** | **Included formulation(s)** | **Conversion factor** |
| Codeine | Capsule, elixir, tablet; sole ingredient and combination products including acetaminophen, butalbital, caffeine | 0.15 |
| Fentanyl | Patch | 7.2 |
| Hydrocodone | Capsule, elixir, tablet; sole ingredient and combination products including acetaminophen, ibuprofen; immediate release and sustained action | 1 |
| Hydromorphone | Suppository, tablet; immediate release and sustained action | 4 |
| Levorphanol | Tablet | 11 |
| Meperidine | Tablet | 0.1 |
| Methadone | Tablet, solution | 3 |
| Morphine | Capsule, solution, suppository, tablet; immediate release and sustained action | 1 |
| Oxycodone | Capsule, solution, tablet; sole ingredient and combination products including acetaminophen; immediate release and sustained action | 1.5 |
| Oxymorphone | Tablet; immediate release and sustained action | 3 |
| Pentazocine | Tablet | 0.37 |
| Tapentadol | Tablet; sustained action | 1 |
| **Non-qualifying opioids** | | |
| **Opioid** | **Non-qualifying formulation(s) and rationale** | **Conversion factor** |
| All | Injectable solutions (indicated for acute or palliative care) | n/a |
| All | Combination products including antitussives or antihistamines (indicated for cough) | n/a |
| Buprenorphine | Patch (added to VA formulary in July 2016) ^a^ | 2.2 |
| Buprenorphine | Sublingual tablets and films (indicated for opioid use disorder) ^a^ | n/a |
| Fentanyl | Lozenge (indicated for cancer or palliative care) | 0.13 |
| Tramadol | All formulations (not expected to be decreased by VA opioid safety initiatives) ^b^ | 0.1 |

1. Buprenorphine was not a qualifying opioid because it was predominantly prescribed for opioid use disorder in VA in 2016.
2. Because tramadol is a weak mu opioid agonist with low potential for abuse (i.e., DEA schedule IV), we did not expect VA initiatives focused on high-risk opioid prescribing practices to have similar effects on tramadol prescribing as on prescribing of stronger schedule II-III opioid analgesics.
