# Supplementary material for: The evaluating prescription opioid changes in veterans (EPOCH) study: Design, survey response, and baseline characteristics
Source: PLoS One. 2020 Apr 22;15(4):e0230751. doi: 10.1371/journal.pone.0230751 (PMC7176145; doi:10.1371/journal.pone.0230751)
Supplement: S2 Table — (DOCX) [file pone.0230751.s002.docx]

**S2 Table:** **Patient exclusion criteria**

| **Exclusion criterion** | **Definition** |
| --- | --- |
| Opioid use disorder treatment | Any outpatient encounter in prior 12 months with a code for Opioid Substitution (523). |
| Dementia | Any outpatient encounter in prior 12 months with a code for Dementia Clinic (320); any problem list or outpatient encounter in the prior 12 months with any of the following diagnosis codes: ICD-9 290, 290.1, 290.11, 290.12, 290.13, 290.2, 290.21, 290.3, 290.4, 290.41, 290.42, 290.43, 290.8, 290.9, 331, 331.1, 331.11, 331.19, 331.2, 331.6, 331.7, 331.82 or ICD-10 F01.5, F01.50, F01.51, F02.80, F02.81, F03.90, F03.91, G30.0, G30.1, G30.8, G30.9, G31.01, G31.09, G31.1,G31.83, R41.81 |
| Cancer treatment | Any outpatient encounter in prior 12 months with a code for Radiation Oncology/Therapy (149), Brachytherapy (158), or Oncology Tumor (316) |
| End-of-life care | Any outpatient encounter in prior 12 months with a code for Hospice Care (351) or Palliative Care (353) |
| Adult day care | Any outpatient encounter in prior 12 months with a code for Adult Day Health Care (190), Community Adult Day Health Care Follow-up (191) |
| Nursing home residence | An inpatient census record without a corresponding discharge; index opioid dispensed between admission and discharge dates for any of the following bed section codes: Respite Care NHCU (47), NH Short Stay Restorative (66), NH Short Stay Continuing Care (67), NH Short Stay Mental Health (68), NH Short Stay Dementia Care (69), Nursing Home Care (80), NH Short Stay Skilled Nursing (95), Hospice (96), Short Stay GRECC NHCU (100), Long Stay GRECC NHCU (101), Short Stay GRECC GEM NHCU (102), or Hospice for Acute Care (105) |
