# Supplementary material for: The evaluating prescription opioid changes in veterans (EPOCH) study: Design, survey response, and baseline characteristics
Source: PLoS One. 2020 Apr 22;15(4):e0230751. doi: 10.1371/journal.pone.0230751 (PMC7176145; doi:10.1371/journal.pone.0230751)
Supplement: S3 Table — (DOCX) [file pone.0230751.s003.docx]

**S3 Table: Pain and mental health diagnosis codes categories**

| **Category** | **Included diagnosis codes (from 12 months before index date)** |
| --- | --- |
| **Pain diagnoses^a^** | |
| Back/spine disorders | 349.39, 353.1, 353.3, 353.4, 720.0, 720.1, 720.2, 720.81, 720.89, 720.9, 721.2, 721.3, 721.41, 721.42, 721.5, 721.6, 721.7, 721.8, 721.9, 721.90, 721.91, 722, 722.1, 722.10, 722.11, 722.2, 722.3, 722.30, 722.31, 722.32, 722.39, 722.5, 722.51, 722.52, 722.6, 722.7, 722.70, 722.72, 722.73, 722.8, 722.80, 722.82, 722.83, 722.9, 722.90,722.92, 722.93, 724.0, 724.00, 724.01, 724.02, 724.03, 724.09, 724.1, 724.2, 724.3, 724.4, 724.5, 724.6, 724.7, 724.70, 724.71, 724.79, 724.8, 724.9, 738.3, 738.4, 738.5, 738.6, 739.2, 739.3, 739.4, 739.5, 756.11, 756.12, 756.13, 756.15, 756.16, 756.17, 756.19, 839.2, 839.20, 839.21, 839.40, 839.41, 839.42, 839.49, 846.0, 846.1, 846.2, 846.3, 846.8, 846.9, 847.1, 847.2, 847.3, 847.4, 847.9,848.5, G54.1, G54.3, G54.4, G96.11, L40.53, M08.1, M25.78, M43.00, M43.04,M43.05, M43.06, M43.07, M43.08, M43.09, M43.10, M43.14, M43.15, M43.16, M43.17, M43.18, M43.19, M43.5X4, M43.5X5, M43.5X6, M43.5X7, M43.5X8, M43.5X9, M43.8X4, M43.8X5, M43.8X6, M43.8X7, M43.8X8, M43.8X9, M43.9, M45.0, M45.4, M45.5, M45.6, M45.7, M45.8, M45.9, M46.00, M46.04, M46.05, M46.06, M46.07, M46.08, M46.09,  M46.1, M46.40, M46.44, M46.45, M46.46, M46.47, M46.48, M46.49, M46.80, M46.84, M46.85, M46.86, M46.87, M46.88, M46.89, M46.90, M46.94, M46.95, M46.96, M46.97, M46.98, M46.99, M47.10, M47.14, M47.15, M47.16, ,M47.20, M47.24, M47.25, M47.26, M47.27, M47.28, M47.814, M47.815, M47.816, M47.817, M47.818, M47.819, M47.894, M47.895, M47.896, M47.897, M47.898, M47.899, M47.9, M48.00, M48.04, M48.05, M48.06, M48.07, M48.08, M48.10, M48.14, M48.15, M48.16, M48.17, M48.18, M48.19, M48.20, M48.24, M48.25, M48.26, M48.27, M48.30, M48.34, M48.35, M48.36, M48.37, M48.38, M48.8X4, M48.8X5, M48.8X6, M48.8X7, M48.8X8, M48.8X9, M48.9, M49.80, M49.84, M49.85, M49.86, M49.87, M49.88, M49.89, M51.04, M51.05, M51.06, M51.14, M51.15, M51.16, M51.17, M51.24, M51.25, M51.26, M51.27, M51.34, M51.35, M51.36, M51.37, M51.44, M51.45, M51.46, M51.47, M51.84, M51.85, M51.86, M51.87, M51.9, M53.2X4, M53.2X5, M53.2X6, M53.2X7, M53.2X8, M53.2X9, M53.3, M53.80, M53.84, M53.85, M53.86, M53.87, M53.88, M53.9, M54.00, M54.04, M54.05, M54.06, M54.07, M54.08, M54.09, M54.10, M54.14, M54.15, M54.16, M54.17, M54.18, M54.30, M54.31, M54.32, M54.40, M54.41, M54.42, M54.5, M54.6, M54.89, M54.9, M62.830, M95.4, M95.5, M96.1, M99.02, M99.03, M99.04, M99.05, M99.12, M99.14, M99.18, M99.53, M99.73, M99.79, M99.82, M99.83, M99.84, M99.85, M99.88, Q67.5, Q76.0, Q76.1, Q76.2, Q76.3, Q76.414, Q76.415, Q76.419, Q76.49, S23.100A, S23.101A, S23.101D, S23.101S, S23.111A, S23.111D, S23.111S, S23.121A, S23.121D, S23.121S, S23.123A, S23.123D, S23.123S, S23.131A, 3.131D, S23.131S, S23.133A, S23.133D, S23.133S, S23.141A, S23.141D, S23.141S, 23.143A, S23.143D, S23.143S, S23.151A, S23.151D, S23.151S, S23.153A, S23.153D, 23.153S, S23.161A, S23.161D, S23.161S, S23.163A, S23.163D, S23.163S, S23.171A, S23.171D, S23.171S, S23.20XA, S23.20XD, S23.20XS, S23.29XA, S23.29XD, S23.29XS, S23.3XXA, S23.3XXD, S23.3XXS, S23.8XXA, S23.8XXD, S23.8XXS, S23.9XXA, S23.9XXD, S23.9XXS, S29.019A, S29.019D, S29.019S, S33.0XXA, S33.100A, S33.101A, S33.101D, S33.101S, S33.111A, S33.111D, S33.111S, S33.121A, S33.121D, S33.121S, S33.131A, S33.131D, S33.131S, S33.140A, S33.140D, S33.141A, S33.141D, S33.141S, S33.2XXA, S33.2XXD, S33.2XXS, S33.30XA, S33.30XD, S33.30XS, S33.39XA, S33.39XD, S33.39XS, S33.5XXA, S33.5XXD, S33.5XXS, S33.6XXA, S33.6XXD, S33.6XXS, S33.8XXA, S33.8XXD, S33.8XXS, S33.9XXA, S33.9XXD, S33.9XXS, S39.012A, S39.012D, S39.012S, S39.92XA |
| Neck/spine disorders | 353.2, 721.0, 721.1, 722.0, 722.4, 722.71, 722.81, 722.91, 723.0, 723.1, 723.2, 723.3, 723.4, 723.5, 723.6, 723.7, 723.8, 723.9, 738.2, 739.1, 839.0, 839.00, 839.01, 839.02, 839.03, 839.04, 839.05, 839.06, 839.07, 839.08, 847.0, 848.2, G54.2, M43.01, M43.02, M43.03, M43.11, M43.12, M43.13, M43.3, M43.4, M43.5X2, M43.5X3, M43.6, M43.8X1, M43.8X2, M43.8X3, M45.1, M45.2, M45.3, M46.01, M46.02, M46.03, M46.41, M46.42, M46.43, M46.81, M46.82, M46.83, M46.91, M46.92, M46.93, M47.11, M47.12, M47.13, M47.22, M47.811, M47.812, M47.813, M47.891, M47.892, M47.893, M48.01, M48.02, M48.03, M48.11, M48.12, M48.13, M48.21, M48.22, M48.23, M48.31, M48.32, M48.33, M48.8X1, M48.8X2, M48.8X3, M49.81, M49.82, M49.83, M50.00, M50.01, M50.020, M50.021, M50.022, M50.023, M50.03, M50.10, M50.11, M50.120, M50.121, M50.122, M50.123, M50.13, M50.20, M50.21, M50.220, M50.221, M50.222, M50.223, M50.23, M50.30, M50.31, M50.320, M50.321, M50.322, M50.323, M50.33, M50.80, M50.81, M50.820, M50.821, M50.822, M50.823, M50.83, M50.90, M50.91, M50.920, M50.921, M50.922, M50.923, M50.93, M53.0, M53.1, M53.2X1, M53.2X2, M53.2X3, M53.81, M53.82, M53.83, M54.01, M54.02, M54.03, M54.11, M54.12, M54.13, M54.2, M95.3, M99.01, M99.11, M99.31, M99.51, M99.61, M99.71, M99.81, Q76.411, Q76.412, Q76.413, S13.0XXA, S13.100A, S13.100D, S13.101A, S13.101D, S13.101S, S13.111A, S13.111D, S13.111S, S13.120A, S13.121A, S13.121D, S13.121S, S13.130A, S13.131A, S13.131D, S13.131S, S13.140A, S13.141A, S13.141D, S13.141S, S13.150A, S13.151A, S13.151D, S13.151S, S13.160A, S13.160D, S13.161A, S13.161D, S13.161S, S13.170D, S13.171A, S13.171D, S13.171S, S13.180A, S13.181A, S13.181D, S13.181S, S13.20XA, S13.20XD, S13.20XS, S13.29XA, S13.29XD, S13.29XS, S13.4XXA, S13.4XXD, S13.4XXS, S13.5XXA, S13.8XXA, S13.8XXD, S13.8XXS, S13.9XXD, S13.9XXS, S16.1XXA, S16.1XXD, S16.1XXS |
| Osteoarthritis | 715, 715.0, 715.00, 715.04, 715.09, 715.1, 715.10, 715.11, 715.12, 715.13, 715.14, 715.15, 715.16, 715.17, 715.18, 715.2, 715.20, 715.21, 715.22, 715.23, 715.24, 715.25, 715.26, 715.27, 715.28, 715.3, 715.30, 715.31, 715.32, 715.33, 715.34, 715.35, 715.36, 715.37, 715.38, 715.8, 715.80, 715.89, 715.9, 715.90, 715.91, 715.92, 715.93, 715.94, 715.95, 715.96, 715.97, 715.98, M15.0, M15.1, M15.2, M15.3, M15.4, M15.8, M15.9, M16.0, M16.10, M16.11, M16.12, M16.2, M16.30, M16.31, M16.32, M16.4, M16.50, M16.51, M16.52, M16.6, M16.7, M16.9, M17.0, M17.10, M17.11, M17.12, M17.2, M17.30, M17.31, M17.32, M17.4, M17.5, M17.9, M18.0, M18.10, M18.11, M18.12, M18.2, M18.30, M18.31, M18.32, M18.4, M18.50, M18.51, M18.52, M18.9, M19.011, M19.012, M19.019, M19.021, M19.022, M19.029, M19.031, M19.032, M19.039, M19.041, M19.042, M19.049, M19.071, M19.072, M19.079, M19.111, M19.112, M19.119, M19.121, M19.122, M19.129, M19.131, M19.132, M19.139, M19.141, M19.142, M19.149, M19.171, M19.172, M19.179, M19.211, M19.212, M19.219, M19.221, M19.222, M19.229, M19.231, M19.232, M19.239, M19.241, M19.242, M19.249, M19.271, M19.272, M19.279, M19.90, M19.91, M19.92, M19.93, M24.7 |
| Neuropathy | 250.60, 250.61, 250.62, 250.63, 357.2, E08.42, E10.40, E10.42, E10.43, E10.610, E11.40, E11.42, E11.43, E11.610, E13.40, E13.42, 053.12, 053.13, 337.0, 337.00,  337.09, 337.1, 350, 350.1, 350.8, 350.9, 352.1, 353.8, 353.9, 354.8, 354.9, 355.79, 355.8, 355.9, 356.4, 356.8, 356.9, 729.2, B02.22, B02.23, E10.41, E13.41, E13.43, G50.0, G50.8, G50.9, G52.1, G54.8, G54.9, G56.80, G56.81, G56.82, G56.83, G56.90, G56.91, G56.92, G56.93, G57.80, G57.81, G57.82, G57.83, G57.90, G57.91, G57.92, G57.93, G58.7, G58.8, G58.9, G59, G60.2, G60.3, G60.8, G60.9, G61.9, G62.0, G62.89, G62.9, G63, G90.09, G99.0, M79.2 |
| Headache | 307.81, 339, 339.0, 339.00, 339.01, 339.02, 339.03, 339.04, 339.05, 339.09, 339.1,  339.10, 339.11, 339.12, 339.2, 339.20, 339.21, 339.22, 339.4, 339.41, 339.42, 339.43, 339.44, 339.8, 339.81, 339.82, 339.83, 339.84, 339.85, 339.89, 346, 346.0, 346.00, 346.01, 346.02, 346.03, 346.1, 346.10, 346.11, 346.12, 346.13, 346.2, 346.20, 346.21, 346.22, 346.23, 346.3, 346.30, 346.31, 346.32, 346.33, 346.4, 346.40, 346.41, 346.42, 346.43, 346.5, 346.50, 346.51, 346.52, 346.53, 346.6, 346.60, 346.61, 346.62, 346.63, 346.7, 346.70, 346.71, 346.72, 346.73, 346.8, 346.80, 346.81, 346.82,346.83, 346.9, 346.90, 346.91, 346.92, 346.93, 784.0, G43.001, G43.009, G43.011, G43.019, G43.101, G43.109,G43.111, G43.119, G43.401, G43.409, G43.411, G43.419, G43.501, G43.509, G43.511, G43.519, G43.601, G43.609, G43.611, G43.619, G43.701, G43.709, G43.711, G43.719, G43.801, G43.809, G43.811, G43.819, G43.821, G43.829, G43.831, G43.839, G43.901, G43.909, G43.911, G43.919, G43.B0, G43.B1, G43.C0, G43.C1, G44.001, G44.009, G44.011, G44.019, G44.021, G44.029, G44.039, G44.049, G44.051, G44.059, G44.099, G44.1, G44.201, G44.209, G44.211, G44.219, G44.221, G44.229, G44.301, G44.309, G44.311, G44.319, G44.321, G44.329, G44.51, G44.52, G44.53, G44.59, G44.81, G44.82, G44.83, G44.84, G44.85, G44.89, M54.81, R51 |
| **Mental health diagnoses^b^** | |
| Depressive disorders | 296.2, 296.21, 296.22, 296.23, 296.24, 296.25, 296.26, 296.3, 296.31, 296.32, 296.33,  296.34, 296.35, 296.36, 300.4, 296.82, 309.1, 311, F32.0, F32.1, F32.2, F32.3, F32.4,  F32.5, F32.9, F33.0, F33.1, F33.2, F33.3, F33.40, F33.41, F33.42, F33.9, F06.31, F06.32, F32.8, F33.8, F34.1 |
| Anxiety disorders | 300, 300.01, 300.02, 300.09, 300.2, 300.21, 300.22, 300.23, 300.29, 293.84, 300.3, F06.4, F40.00, F40.01, F40.02, F40.10, F40.11, F40.210, F40.218, F40.220, F40.228, F40.230, F40.231, F40.232, F40.233, F40.240, F40.241, F40.242, F40.243, F40.248, F40.290, F40.291, F40.298, F40.8, F40.9, F41.0, F41.1, F41.3, F41.8, F41.9, F42, F45.20, F45.21, F45.29 |
| PTSD | 309.81, F43.10, F43.11, F43.12 |
| Alcohol use disorder | 291.81, 291.89, 291.9, 303, 303.01, 303.02, 303.03, 303.9, 303.91, 303.92, 303.93,  305, 305.01, 305.02, 305.03, F10.27, F10.10, F10.120, F10.121, F10.129, F10.14, F10.150, F10.151, F10.159, F10.180, F10.181, F10.182, F10.188, F10.19, F10.20, F10.21, F10.220, F10.221, F10.229, F10.230, F10.231, F10.232, F10.239, F10.24, F10.250, F10.251, F10.259, F10.26, F10.280, F10.281, F10.282, F10.288, F10.29, F10.920, F10.921, F10.929, F10.94, F10.950, F10.951, F10.959, F10.96, F10.97, F10.980, F10.981, F10.982, F10.988, F10.99 |
| Drug use disorders | 305.2, 305.21, 305.22, 305.23, 305.3, 305.31, 305.32, 305.33, 305.4, 305.41, 305.42, 305.43, 305.5, 305.51, 305.52, 305.53, 305.6, 305.61, 305.62, 305.63, 305.7, 305.71, 305.72, 305.73, 305.8, 305.81, 305.82, 305.83, 305.9, 305.91, 305.92, 305.93, 304, 304.01, 304.02, 304.03, 304.1, 304.11, 304.12, 304.13, 304.2, 304.21, 304.22, 304.23, 304.3, 304.31, 304.32, 304.33, 304.4, 304.41, 304.42, 304.43, 304.5, 304.51, 304.52, 304.53, 304.6, 304.61, 304.62, 304.63, 304.7, 304.71, 304.72, 304.73, 304.8, 304.81, 304.82, 304.83, 304.9, 304.91, 304.92, 304.93, F19.97, F11.10, F11.120, F11.121, F11.122, F11.129, F11.14, F11.150, F11.151, F11.159, F11.181, F11.182, F11.188, F11.19, F11.20, F11.21, F11.220, F11.221, F11.222, F11.229, F11.23, F11.24, F11.250, F11.251, F11.259, F11.281, F11.282, F11.288, F11.29, F11.90, F11.920, F11.921, F11.922, F11.929, F11.93, F11.94, F11.950, F11.951, F11.959, F11.981, F11.982, F11.988, F11.99, F12.10, F12.120, F12.121, F12.122, F12.129, F12.150, F12.151, F12.159, F12.180, F12.188, F12.19, F12.20, F12.21, F12.220, F12.221, F12.222, F12.229, F12.250, F12.251, F12.259, F12.280, F12.288, F12.29, F12.90, F12.920, F12.921, F12.922, F12.929, F12.950, F12.951, F12.959, F12.980, F12.988, F12.99, F13.10, F13.120, F13.121, F13.129, F13.14, F13.150, F13.151, F13.159, F13.180, F13.181, F13.182, F13.188, F13.19, F13.20, F13.21, F13.220, F13.221, F13.229, F13.230, F13.231, F13.232, F13.239, F13.24, F13.250, F13.251, F13.259, F13.26, F13.27, F13.280, F13.281, F13.282, F13.288, F13.29, F13.90, F13.920, F13.921, F13.929, F13.930, F13.931, F13.932, F13.939, F13.94, F13.950, F13.951, F13.959, F13.96, F13.97, F13.980, F13.981, F13.982, F13.988, F13.99, F14.10, F14.120, F14.121, F14.122, F14.129, F14.14, F14.150, F14.151, F14.159, F14.180, F14.181, F14.182, F14.188, F14.19, F14.20, F14.21, F14.220, F14.221, F14.222, F14.229, F14.23, F14.24, F14.250, F14.251, F14.259, F14.280, F14.281,F14.282, F14.288, F14.29, F14.90, F14.920, F14.921, F14.922, F14.929, F14.94, F14.950, F14.951, F14.959, F14.980, F14.981, F14.982, F14.988, F14.99, F15.10, F15.120, F15.121, F15.122, F15.129, F15.14, F15.150, F15.151, F15.159, F15.180, F15.181, F15.182, F15.188, F15.19, F15.20, F15.21, F15.220, F15.221, F15.222, F15.229, F15.23, F15.24, F15.250, F15.251, F15.259, F15.280, F15.281, F15.282, F15.288, F15.29, F15.90, F15.920, F15.921, F15.922, F15.929, F15.93, F15.94, F15.950, F15.951, F15.959, F15.980, F15.981, F15.982, F15.988, F15.99, F16.10, F16.120, F16.121, F16.122, F16.129, F16.14, F16.150, F16.151, F16.159, F16.180, F16.183, F16.188, F16.19, F16.20, F16.21, F16.220, F16.221, F16.229, F16.24, F16.250, F16.251, F16.259, F16.280, F16.283, F16.288, F16.29, F16.90, F16.920, F16.921, F16.929, F16.94, F16.950, F16.951, F16.959, F16.980, F16.983, F16.988, F16.99, F18.10, F18.120, F18.121, F18.129, F18.14, F18.150, F18.151, F18.159, F18.17, F18.180, F18.188, F18.19, F18.20, F18.21, F18.220, F18.221, F18.229, F18.24, F18.250, F18.251, F18.259, F18.27, F18.280, F18.288, F18.29, F18.90, F18.920, F18.921, F18.929, F18.94, F18.950, F18.951, F18.959, F18.97, F18.980, F18.988, F18.99, F19.10, F19.120, F19.121, F19.122, F19.129, F19.14, F19.150, F19.151, F19.159, F19.16, F19.17, F19.180, F19.181, F19.182, F19.188, F19.19, F19.20, F19.21, F19.220, F19.221, F19.222, F19.229, F19.230, F19.231, F19.232, F19.239, F19.24, F19.250, F19.251, F19.259, F19.26, F19.27, F19.280, F19.281, F19.282, F19.288, F19.29, F19.90, F19.920, F19.921, F19.922, F19.929, F19.930, F19.931, F19.932, F19.939, F19.94, F19.950, F19.951, F19.959, F19.96, F19.980, F19.981, F19.982, F19.988, F19.99 |

a) Pain diagnosis code categories based on work of musculoskeletal diagnosis cohort investigators (Goulet JL, Kerns RD, Bair M, Becker WC, Brennan P, et al. The musculoskeletal diagnosis cohort: examining pain and pain care among veterans. Pain 2016;157(8):1696-1703.)

b) Mental health diagnosis code categories based on work of Program Evaluation and Resource Center, VHA Office of Mental Health and Suicide Prevention.
