# Supplementary material for: The evaluating prescription opioid changes in veterans (EPOCH) study: Design, survey response, and baseline characteristics
Source: PLoS One. 2020 Apr 22;15(4):e0230751. doi: 10.1371/journal.pone.0230751 (PMC7176145; doi:10.1371/journal.pone.0230751)
Supplement: S4 Table — (DOCX) [file pone.0230751.s004.docx]

**S4 Table: Analysis of response among eligible patients selected for invitation (n=13,976)^a^**

|  |  | **Series 1** | **Series 2** | **Series 3** | **Series 4** | **Series 5** | **Series 6** | **Series 7** | **Series 8** | **Series 9** | **Series 10** | **Series 11** | **Series 12** |
| --- | --- | --- | --- | --- | --- | --- | --- | --- | --- | --- | --- | --- | --- |
| **Variable ^b^** | All patients | 35 random sets of distinct 400 except last set is 376 | 24 random sets of distinct 600 except last set is 176 | 18 random sets of distinct 800 except last set is 376 | 14 random sets of distinct 1000 except last set is 976 | 7 random sets of distinct 2000 except last set is 1976 | 5 random sets of distinct 3000 except last set is 1976 | 4 random sets of distinct 4000 except last set is 1976 | 3 random sets of distinct 5000 except last set is 3976 | 3 random sets of distinct 5000 except last set is 3976, different random seed #190123 | 24 random sets of distinct 600 except last set is 176, different random seed #190123 | 35 random sets of distinct 400 except last set is 376, different random seed #190123 | 35 random sets of distinct 400 except last set is 376, different random seed #190124 |
| Hispanic ethnicity | p=0.0476, Beta= -0.1730, se=0.0873 | ns | ns | ns | ns | ns | ns | ns | ns | ns | ns | ns | ns |
| Post-9/11 military service | p=0.0011, Beta= -0.2736, se=0.0842 | ns | ns | ns | ns | ns | ns | ns | ns | p=0.0109, Beta= -0.2746 se=0.0981 | ns | ns | ns |
| Urban residence | p=0.0006, Beta= -0.1268 se=0.0369 | ns | ns | ns | ns | ns | ns | ns | ns | ns | ns | ns | ns |
| Back/ spine disorder | p=0.0002, Beta= 0.1489, se=0.0397 | ns | ns | ns | ns | ns | ns | ns | ns | ns | ns | ns | ns |
| Neck/ spine disorders | p=0.0455, Beta= 0.0909, se=0.0454 | ns | ns | ns | ns | ns | ns | ns | ns | ns | ns | ns | ns |
| Osteo-arthritis | p=0.0133, Beta= 0.1015, se=0.0410 | ns | ns | ns | ns | ns | ns | ns | ns | ns | ns | ns | ns |
| Neuro-pathy | p=0.0134, Beta= 0.1186, se=0.0480 | ns | ns | ns | ns | ns | ns | ns | ns | ns | ns | ns | ns |
| Depres-sion disorder | p<.0001, Beta= 0.2347, se=0.0428 | ns | ns | ns | ns | p=0.02070, Beta= 0.2345, se=0.0856 | p=0.0240, Beta= 0.2388, se=0.0831 | p=0.0175, Beta= 0.2432, se=0.0760 | p<.0001, Beta= 0.2383, se=0.0483 | ns | ns | ns | ns |
| Anxiety disorder | p=0.0194, Beta= -0.1211, se=0.0518 | ns | ns | ns | ns | ns | ns | ns | p=0.0249, Beta= -0.1208, se=0.0537 | ns | ns | ns | ns |
| Age | p<.0001, Beta= 0.3391, se=0.0213 | p=0.0459, Beta= 0.3515, se=0.1699 | p=0.0256, Beta= 0.3540, se=0.1488 | p=0.0221, Beta= 0.3436, se=0.1371 | p=0.0100, Beta= 0.3448, se=0.1156 | p=0.0029, Beta= 0.3387, se=0.0754 | p=0.0021, Beta= 0.3337, se=0.0583 | p=0.0136, Beta= 0.3300, se=0.0722 | p<.0001, Beta= 0.3368, se=0.0252 | p=0.0038, Beta= 0.3432, se=0.0435 | p=0.0040, Beta= 0.3573, se=0.1126 | p=0.0187, Beta= 0.3599, se=0.1440 | p=0.0087, Beta= 0.3532, se=0.1273 |
| Average pain score in prior year | p=0.0222, Beta= -0.0431 se=0.0188 | ns | ns | ns | ns | ns | ns | ns | ns | ns | ns | ns | ns |

Abbreviations: Beta = beta coefficient from logistic regression model; ns = not significant at p<0.05 level; se = standard error.

The table shows results of separate logistic regression models including 11 baseline variables in different datasets. Three sets of random seed generators were used to create series of distinct and independent datasets of sizes 400 to 1000 (with increments of 200) and 1000 to 5000 (with increments of 1000).

a) 13,976 included in analysis were 14,160 selected for invitation to participate minus 184 who did not have complete data on all 24 variables. Missing variables were as follows: 174 missing average pain score; 60 missing VA enrollment priority group; 15 missing both distance to VA facility and drive time to VA facility (of whom 8 were also missing VA enrollment priority group).

b) The 24 baseline variables were the following: Age; sex (male, female); race (white, black, other); Hispanic ethnicity (yes, no, unknown); married (yes, no); urban residence (yes, no); VA enrollment priority group (1-4, 5-6, 7-8); distance to VA facility; drive time to VA facility; post-9/11 military service (yes, no); average pain score in prior year; 5 pain diagnosis categories (back/spine, neck/spine, osteoarthritis, neuropathy, headache; 6 mental health diagnosis categories (depressive disorder, anxiety disorder, PTSD, alcohol use disorder, drug use disorder, opioid use disorder; Charlson comorbidity score; opioid daily dosage. Data for 13 variables not significant at p<0.05 level in initial logistic regression model are not shown.
