# Supplementary material for: The evaluating prescription opioid changes in veterans (EPOCH) study: Design, survey response, and baseline characteristics
Source: PLoS One. 2020 Apr 22;15(4):e0230751. doi: 10.1371/journal.pone.0230751 (PMC7176145; doi:10.1371/journal.pone.0230751)
Supplement: S5 Table — (DOCX) [file pone.0230751.s005.docx]

**S5 Table: Relationship of opioid daily dosage in 10 mg increments with outcome measures in patients treated with and without long-acting opioids.**

| **Outcome** | **Sample** | **Beta coefficient** | **SE** | **p-value** |
| --- | --- | --- | --- | --- |
| BPI-I (n=8956) | No long-acting opioid (n=6408) | 0.096 | 0.014 | <0.0001 |
|  | No long-acting opioid and daily dosage limited to <200 ME mg (n=6398)^a^ | 0.097 | 0.015 | <0.0001 |
|  | Long-acting opioid (n=2548) | 0.013 | 0.007 | 0.0681 |
|  | Long-acting opioid and daily dosage limited to <500 ME mg (n=2530) ^a^ | 0.021 | 0.008 | 0.0121 |
| VR-12 PCS (n=8777) | No long-acting opioid (n=6286) | -0.408 | 0.061 | <0.0001 |
|  | No long-acting opioid and daily dosage limited to <200 ME mg (n=6275) ^a^ | -0.441 | 0.070 | <0.0001 |
|  | Long-acting opioid (n=2491) | -0.0395 | 0.034 | 0.2410 |
|  | Long-acting opioid and daily dosage limited to <500 ME mg (n=2472) ^a^ | -0.078 | 0.038 | 0.0414 |
| VR-12 MCS (n=8763) | No long-acting opioid (n=6276) | -0.393 | 0.107 | 0.0003 |
|  | No long-acting opioid and daily dosage limited to <200 ME mg (n=6265) ^a^ | -0.405 | 0.125 | 0.0012 |
|  | Long-acting opioid (n=2487) | -0.092 | 0.041 | 0.0236 |
|  | Long-acting opioid and daily dosage limited to <500 ME mg (n=2468) ^a^ | -0.120 | 0.053 | 0.0231 |

Data are from age-adjusted weighted linear regression models including patients with complete data for each outcome.

a) Sensitivity analysis with dosage-restricted sample.
