# Supplementary material for: The evaluating prescription opioid changes in veterans (EPOCH) study: Design, survey response, and baseline characteristics
Source: PLoS One. 2020 Apr 22;15(4):e0230751. doi: 10.1371/journal.pone.0230751 (PMC7176145; doi:10.1371/journal.pone.0230751)
Supplement: Attachment — Submitted filename: Response to Reviewers.docx [file pone.0230751.s006.docx]

PONE-D-19-31436

Response to Reviewers

**Editorial comments:**

1. *Please ensure that your manuscript meets PLOS ONE's style requirements, including those for file naming.*

We have applied style requirements, including those for file names.

2. *Please ensure you have thoroughly discussed any potential limitations of this study within the Discussion section, including the potential bias introduced by using self-reported data.*

We added limitations related to self-report and administrative data (page 22, lines 486-491).

*3. Please further describe how the "minimum panel size criteria" was calculated.*

We added details about our approach to identifying primary care providers and applying minimum panel size criteria. To improve clarity for readers, we put this information in a new methods subsection, “survey sample selection.” (pages 6-7, lines 145-166)

*4. Please upload a copy of Figure 3, to which you refer in your text.*

Figure 3 is included with the submission.

*5. Please include captions for your Supporting Information files at the end of your manuscript, and update any in-text citations to match accordingly.*

Supporting information captions are now included at the end of the manuscript and in-text citations are updated to match. (pages 28-29, lines 631-637)

**Reviewer #1 comments:**

*1. In table 1, it shows back/spine disorder is the most commonly pain type in the survey. Is there special reason leading to massive back/spine injuries among veterans?*

We added information about back pain prevalence to the discussion. (age 20, lines 434-438)

*2. This survey has a very good response rate. What would the author think is the most important contributor?*

We used multiple methods that have been found to improve response rates or that we hoped would help, so we can’t disentangle the most important reasons for our success. I added to the discussion an additional contributor—the ongoing clinical relationship study patients had with VA clinics. (page 19, lines 418-421)

*3. I am personally very curious about what can be a potential alternative for opioid?*

Although care should be individualized, guidelines recommend a variety of other medications, exercise therapies, psychological therapies such as cognitive behavioral therapy, manual treatments such as spinal manipulation, and mind-body approaches such as yoga.

**Reviewer #2 comments:**

*1. Please organize the tables, it's way too busy, very hard to went through, better split to 2-3 different tables.*

We split Tables 1 into two tables, separating patient characteristics from opioid treatment received. (Pages 12 and 13) I am unsure of a better way to present data in the other tables but am open to specific suggestions you may have.

*2. If Fig 1 is using the table version for presenting, please rename as Table*

Fig 1 is the study flow diagram and is not in table format.
